# Neck Swelling After Total Parenteral Nutrition

**DOI:** 10.1016/j.acepjo.2026.100346

**Published:** 2026-02-26

**Authors:** Nicholas Maffetone, Emma Alley, Bradley E. Brocious

**Affiliations:** Department of Emergency Medicine, Geisinger Medical Center, Danville, Pennsylvania, USA

**Keywords:** TPN, PICC, Mediastinitis, Bacteremia, CLABSI

## 1 Case Presentation

A 72-year-old woman with severe malnutrition on total parenteral nutrition (TPN) through a left upper extremity peripherally inserted central catheter (PICC) line presented to the emergency department (ED) with 4 hours of swelling around her neck and chest after initiating feeds. She reported one episode of fever the day prior. She was hemodynamically stable. Physical examination revealed swelling of the chest and anterior neck with palpable fluctuance, without erythema or crepitus. Laboratory results demonstrated leukocytosis, elevated procalcitonin, and C-reactive protein levels. Blood cultures were obtained. Computed tomography scans of the neck and chest were obtained (Fig).

**Figure:**
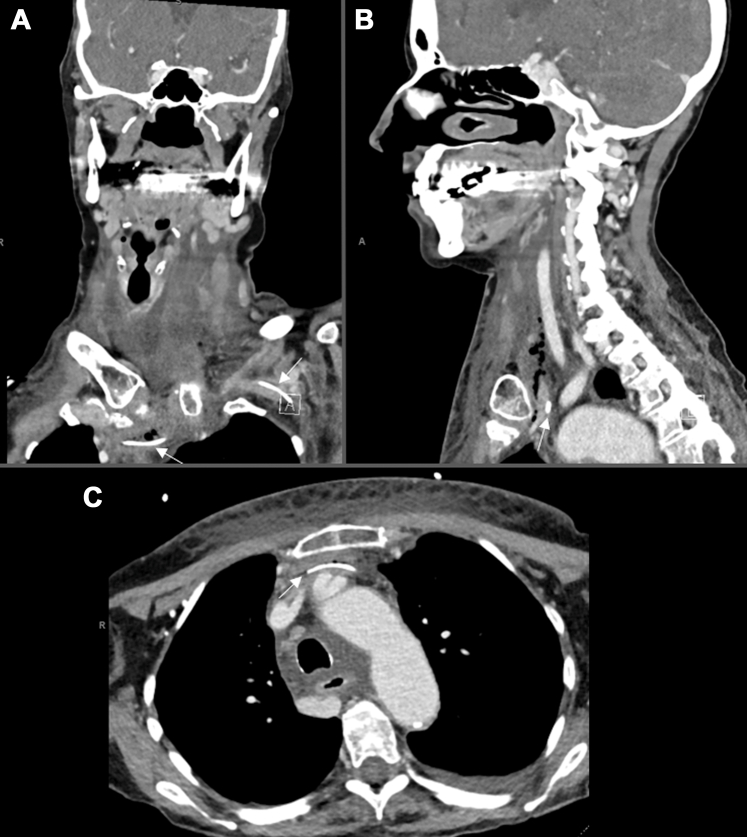
Computed tomography scan shows left upper extremity peripherally inserted central catheter (PICC) with tip terminating anterior to the left brachiocephalic vein (bottom arrow in A and arrow in C). Arrows are pointing to the PICC line. The top arrow in A shows the PICC line within the brachiocephalic vein. The tip of the PICC line is approximately 2 cm anterior to the confluence in the anterior mediastinum, with pneumomediastinum ascending along the left internal jugular vein (arrow in B) and low-density fluid in stranding throughout the anterior mediastinum, at the anterior chest subcutaneous soft tissues, and the deep spaces of the neck tracking superiorly.

## 2 Diagnosis: PICC Mispositioning Causing Pneumomediastinum, Mediastinitis, and Bacteremia

In this case, the PICC line tip was exiting the left brachiocephalic vein, therefore infiltrating TPN into the mediastinum. The patient was started on antibiotics and antifungal drugs in the ED and admitted. *Staphyllococcus epidermidis* was isolated from blood cultures. The patient’s clinical course was complicated by septic thrombophlebitis, left cerebellar septic emboli, and seizure. Overall, this case highlighted the importance of early recognition of PICC line complications in the ED. PICC lines have high rates of complications, including occlusion, accidental withdrawal, infection, and venothrombosis.^1^ Infiltration from a PICC line commonly occurs shortly after placement; however, this complication may present later in the lifespan of the PICC line if it erodes through the vessel. In this case, the PICC line was placed almost a year prior to the ED encounter. Prompt recognition, collection of cultures, initiation of antibiotics, and stopping use of the PICC line are critical in the management of TPN infiltration into the mediastinum from a PICC line.

## Funding and Support

By *JACEP Open* policy, all authors are required to disclose any and all commercial, financial, and other relationships in any way related to the subject of this article as per ICMJE conflict of interest guidelines (see www.icmje.org). The authors have stated that no such relationships exist.

## Conflict of Interest

All authors have affirmed they have no conflicts of interest to declare.
